# Supplementary material for: Quantitative characterization of biological age and frailty based on locomotor activity records
Source: Aging (Albany NY). 2018 Oct 25;10(10):2973–90. doi: 10.18632/aging.101603 (PMC6224248; doi:10.18632/aging.101603)
Supplement: APPENDIX [file aging-10-101603-s001.pdf]

SUPPLEMENTARY MATERIAL

APPENDIX

A. Transition matrix and Power Spectrum Density

Under the Markov chain model, the evolution of the probability $P_i(t)$ to find the system at state i for the system with N discrete states is governed by the master equation which in the linear mode can be written as

$$\frac{dP_i(t)}{dt} = \sum_{j=1}^{N} \left(k_{ij}P_j(t) - k_{ji}P_i(t)\right), \qquad (1)$$

where $k_{ij}\geq 0$ is the rate of transition from state j to state i. By introducing the transition matrix (TM) according to

$$W_{ij} = k_{ij} - \delta_{ij}\sum_{e=1}^{N} k_{ei}, \qquad (2)$$

we can rewrite Eq. 1 as

$$P_i(t) = \sum_{j=1}^{N} W_{ij}P_j(t). \qquad (3)$$

Note from Eq. 2 and definition of k_{ij} we have $W_{ij}\geq 0$ for $i\neq j$, $W_{ii}\leq 0$ and

$$\sum_{i=1}^{N} W_{ij} = 0, \qquad (4)$$

from which it follows that the probability norm is preserved $d/dt(\Sigma P_i)=0$, as it should be.

In the following analysis we will assume that the TM W is irreducible and has distinct eigenvalues. The reasoning for such assumptions will be provided later. Under this assumptions W can be diagonalized:

$$W_{ij} = \sum_{k=1}^{N} \lambda_k A_{kj}B_{ki}, \qquad (5)$$

Where A_k and B_k are left ($\Sigma_i W_{ij}A_{ki}=\lambda_k A_{kj}$) and right ($\Sigma_j W_{ij}B_{kj}=\lambda_k B_{ki}$) eigenvectors corresponding to eigenvalue λ_k. Note that the systems of left and right eigenvectors are the inverse for each other:

$$\sum_{k=1}^{N} A_{ki}B_{kj} = \delta_{ij} \; and \; \sum_{i=1}^{N} A_{ki}B_{mi} = \delta_{km}. \qquad (6)$$

To solve Eq. 3 we introduce

$$Q_k(t) = \sum_{j=1}^{N} A_{kj}P_j(t), \qquad (7)$$

and using Eqs. 5 and 6 rewrite Eq. 3 as

$$Q_k(t) = \lambda_k Q_k(t),$$

for which the solution is

$$Q_k(t) = Q_k(0)e^{\lambda_k t},$$

from which using Eqs. 6 and 7 we get

$$P_i(t) = \sum_{k=1}^{N}\sum_{j=1}^{N} A_{kj}B_{ki}e^{\lambda_k t}P_j^0 = \sum_{j=1}^{N} G_{ij}(t)\,P_j^0, \qquad (8)$$

$$G_{ij}(t) = \sum_{k=1}^{N} A_{kj}B_{ki}e^{\lambda_k t}, \qquad (9)$$

where $G_{ij}(t)$ is the probability $P(i,t|j,0)$ to find the system in state i at time t if the system originally was in state j at time 0 and P^0 is the initial distribution.

The assumption that W has distinct eigenvalues together with Eq. 4 imply that W has exactly one zero eigenvalue. Since the order of eigenvalues is arbitrary, we can state that

$$\begin{cases} \lambda_1 = 0, \\ Re\;\lambda_i < 0, \; 1 < i \leq N \end{cases} \qquad (10)$$

where the later inequality follows from W being a TM. Indeed, W is real-valued, therefore for any eigenvalue λ_i and corresponding left eigenvector A_i we have

$$\lambda_i A_{ij} = \sum_{k=1}^{N} W_{kj}A_{ik},$$

and

$$\lambda_i^* A_{ij}^* = \sum_{k=1}^{N} W_{kj}A_{ik}^*,$$

where * denotes complex conjugate. After multiplying the first equation by A_{ij}*, the second by A_{ij} and summing we get

$$2\,Re\lambda_i \cdot |A_{ij}|^2 = \sum_{k\neq j} W_{kj}\left(A_{ik}A_{ij}^* + A_{ik}^*A_{ij}\right) + 2W_{jj}|A_{ij}|^2.$$

Representing all A_{ij} in exponential form $A_{ij}=\rho_{ij}exp(i\varphi_{ij})$, dividing by $|A_{ij}|^2$ and replacing W_{jj} using Eq. 4 we get

$$Re\lambda_i = \sum_{k\neq j} W_{kj}\left[\frac{\rho_{ik}}{\rho_{ij}} cos\left(\varphi_{ik} - \varphi_{ij}\right)^{-1}\right]. \qquad (11)$$

This equation holds for all i and j. For a given i let us choose a particular j such that $\rho_{ik}\leq\rho_{ij}$. Since all ρ_{ik} and W_{kj} for $k\neq j$ are non-negative by definition, the Eq. 11 becomes $Re\lambda_i\leq 0$.

According to Eq. 3, any equilibrium state is the right eigenvector corresponding to the zero eigenvalue. Since W has only one such eigenvector (up to scaling), we have a unique equilibrium distribution given by

$$P_i^{eq} = B_{1i} \Big/ \sum_{j=1}^{N} B_{1j} \qquad (12)$$

The eigensystem has several interesting properties. From Eq. 9 and 10 we get $G_{ij}(+\infty)=A_{1j}B_{1i}$ and the distribution at $t=+\infty$ is

$$P_i^{\infty} = B_{1i} \sum_{j=1}^{N} A_{1j} P_j^0 . \qquad (13)$$

For any initial distribution P^0 the corresponding P^{∞} is an equilibrium state:

$$\sum_{j=1}^{N} G_{ij}(t)\, P_j^{\infty} = \sum_{j=1}^{N}\sum_{k=1}^{N}\sum_{m=1}^{N} A_{kj} B_{ki} e^{\lambda_k t} B_{1j} A_{1m} P_m^0 = \sum_{m=1}^{N} B_{1i} A_{1m} P_m^0 = P_i^{\infty},$$

and since equilibrium is unique $P_i^{\infty} = P_i^{eq}$ for any P^0. From this and Eq. 13 we have

$$A_{1i} = const = 1 \Big/ \sum_{j=1}^{N} B_{1j} . \qquad (14)$$

Using Eq. 4, for the right eigenvectors B_k we get

$$\lambda_k \sum_{i=1}^{N} B_{ki} = \sum_{i=1}^{N}\sum_{j=1}^{N} W_{ij} B_{kj} = 0,$$

and therefore

$$\sum_{i=1}^{N} B_{ki} = 0 \;\; for \;\; \lambda_k \neq 0. \qquad (15)$$

Let as consider a discrete real-valued stochastic process $x(t)$ having value x_i when the system happens to be in state i. According to the Wiener–Khinchin theorem, the power spectral density $S_x(\omega)$ for the $x(t)$ is the Fourier transform of the autocorrelator

$$R_{xx}(t) = \lim_{T\to+\infty} \frac{1}{T} \int_0^T E[x(t+\tau)\, x(t)] dt. \qquad (16)$$

Using the fact that $R_{xx}(\tau)$ is an even real-valued function we obtain

$$S_x(\omega) = 2 \int_0^{+\infty} R_{xx}(\tau) \cos(\omega\tau)\, d\tau. \qquad (17)$$

Here we follow the common physical convention that the total power of the signal is given by $\int_{-\infty}^{+\infty} S_x(\omega) \frac{d\omega}{2\pi}$.

Expanding the Eq. 16 we get

$$R_{xx}(\tau) = \lim_{T\to+\infty} \frac{1}{T} \int_0^T \sum_{i=1}^{N}\sum_{j=1}^{N} x_i\, P(i, t+\tau \,|\, j, t)\, x_j\, P(j,t) dt, \qquad (18)$$

Where $P(i,t+\tau|j,t)$ is the probability to find the system in state j at time $t+\tau$ if the system was in state j at time t and $P(i,t)$ is the probability to find the system in state j at time t, with the evolution of the system starting from some state P^0. From the definitions we have $P(i,t+\tau|j,t)=G_{ij}(\tau)$ for $\tau \geq 0$ and $P(j,t)=P_j(t)$. Using this and Eq. 8 and 9 rewrite Eq. 18 as

$$R_{xx}(\tau) = \lim_{T\to+\infty} \frac{1}{T} \int_0^T \sum_{i=1}^{N}\sum_{j=1}^{N} x_i A_{kj} B_{ki} e^{\lambda_k \tau} x_j A_{mn} B_{mj} e^{\lambda_m t} P_n^0 dt,$$

where $\tau>0$ and the summation is done for each index from 1 to N. By rearranging and using Eq. 10 we get

$$R_{xx}(\tau) = \sum_{i,j,k,n} x_i A_{kj} B_{ki} e^{\lambda_k \tau} x_j A_{1n} B_{1j} P_n^0,$$

from which using Eq. 12 we finally obtain

$$R_{xx}(\tau) = \sum_{i=1}^{N}\sum_{j=1}^{N}\sum_{k=1}^{N} x_i x_j A_{kj} B_{ki} P_j^{eq} e^{\lambda_k \tau}, \quad for \;\; \tau \geq 0. \qquad (19)$$

Note that $R_{xx}(\tau)$ is not dependent on the initial distribution P^0, as it is expected for the system with equilibrium state. The integration of Eq. 19 using Eq. 17 is straightforward, and we get

$$S_x(\omega) = -2 \sum_{k=2}^{N} \left\{ \frac{\lambda_k}{\lambda_k^2 + \omega^2} \sum_{i=1}^{N} x_i A_{ki} P_i^{eq} \sum_{j=1}^{N} x_j B_{kj} \right\}. \qquad (20)$$

The Eq. 20 is valid for any irreducible diagonalizable TM W. In particular, some λ_k may be complex. However, for the real-valued matrix W complex eigenvalues and corresponding eigenvectors always comes in complex conjugate pairs, which, together with Eq. 10, imply that $S_x(\omega)$ is always real positive, as any PSD should be.

Due to time symmetry of the fundamental physical laws, for the systems in thermodynamic equilibrium the detailed balance assumption is hold:

$$W_{ij} P_j^{eq} = W_{ji} P_i^{eq}. \qquad (21)$$

Biological organisms as a whole are not systems in thermodynamic equilibrium and the description of the motion using Markov chain model is only a rough

approximation, so there are no *a priori* reasons to assume the detailed balance. However, experimentally the correlation between $W_{ij}P_j^{eq}$ and $W_{ji}P_i^{eq}$ is good, so it is interesting to see how $S_x(\omega)$ looks under detailed balance assumption.

First we introduce a derived matrix

$$\widetilde{w}_{ij} = W_{ij}\sqrt{P_j^{eq}/P_i^{eq}}. \qquad (22)$$

With Eq. 21 hold, $\widetilde{w}$ is symmetric and therefore can be eigendecomposed into

$$\widetilde{w}_{ij} = \sum_{k=1}^{N} \lambda_k \mu_{ki}\mu_{kj}, \qquad (23)$$

where all eigenvalues λ_k are real and eigenvectors μ_k are orthonormal:

$$\sum_{k=1}^{N} \mu_{ki}\mu_{kj} = \delta_{ij} \quad and \quad \sum_{i=1}^{N} \mu_{ki}\mu_{mi} = \delta_{km}. \qquad (24)$$

From Eqs. 22 and 23 we get

$$W_{ij} = \sqrt{\frac{P_i^{eq}}{P_j^{eq}}}\sum_{k=1}^{N} \lambda_k \mu_{ki}\mu_{kj} = \sum_{k=1}^{N} \lambda_k \tilde{A}_{kj}\tilde{B}_{ki}, \qquad (25)$$

where

$$\tilde{A}_{kj} = \mu_{kj}/\sqrt{P_j^{eq}} \quad and \quad \tilde{B}_{ki} = \mu_{ki}/\sqrt{P_i^{eq}}, \qquad (26)$$

from which using Eq. 24 follows

$$\sum_{k=1}^{N} \tilde{A}_{ki}\tilde{B}_{kj} = \delta_{ij} \quad and \quad \sum_{i=1}^{N} \tilde{A}_{ki}\tilde{B}_{mi} = \delta_{km},$$

which imply that Eq. 25 is an eigendecomposition of W as in Eq. 5, so we can use Eq. 20, which becomes

$$S_x(\omega) = -2\sum_{k=2}^{N} \frac{\lambda_k}{\lambda_k^2 + \omega^2}\left(\sum_{i=1}^{N} x_i \tilde{B}_{ki}\right)^2. \qquad (27)$$

Here we used $\tilde{A}_{ki}=\tilde{B}_{ki}/Pi^{eq}$, obtained from Eq. 26, to express $S_x(\omega)$ via right eigenvectors $\tilde{B}_k$ alone. Each of the right eigenvectors B_k is defined up to a multiplication constant, however the scaling is fixed for $\tilde{B}_k$: from Eqs. 24 and 26 we have

$$\sum_{i=1}^{N} \frac{\tilde{B}_{ki}\tilde{B}_{mi}}{P_i^{eq}} = \delta_{km}, \qquad (28)$$

from which we can find a proper scaling for an arbitrary right eigenvector B_k:

$$\tilde{B}_{ki} = B_{ki}\left(\sum_{j=1}^{N} \frac{\left(B_{kj}\right)^2}{P_j^{eq}}\right)^{-\frac{1}{2}}. \qquad (29)$$

The $S_x(\omega)$ can be calculated under detailed balance assumption as follows: calculate the right eigensystem for W, scale the found eigenvectors B_k using Eq. 29 and finally calculate $S_x(\omega)$ using Eq. 27. The same procedure can be applied when the detailed balance assumption holds only approximately, as long as we drop the imaginary part of the found eigenvalues and right eigenvectors. Note that even when all eigenvalues are real, the Eq. 27 is not equivalent to Eq. 20 without the detailed balance assumption. In particular, scaling according to Eq. 29 is not enough for Eq. 28 to hold, which is required for Eq. 27 to be precise.
